# ESTRO vision 2030: the young Italian Association of Radiotherapy and Clinical Oncology (yAIRO) commitment statement

**DOI:** 10.1007/s11547-021-01398-w

**Published:** 2021-07-20

**Authors:** Francesca De Felice, Luca Boldrini, Carlo Greco, Valerio Nardone, Viola Salvestrini, Isacco Desideri

**Affiliations:** 1 Department of Radiotherapy, Policlinico Umberto I “Sapienza” University of Rome, Viale Regina Elena 326, 00161 Rome, Italy; 2 Fondazione Policlinico Universitario “Agostino Gemelli” IRCCS, Largo Agostino Gemelli, 8, 00168 Rome, Italy; 3 Radiation Oncology, Campus Bio-Medico University, Rome, Italy; 4 Radiotherapy Unit, “Ospedale del Mare”, ASL Napoli 1, 80147 Naples, Italy; 5 Radiation Oncology, Azienda Ospedaliero-Universitaria Careggi, University of Florence, Florence, Italy

**Keywords:** ESTRO, AIRO, Vision, Young, Radiotherapy, Oncology, Strategy

## Abstract

The aim of this document is to share the action plan from the young Italian Association of Radiotherapy and Clinical Oncology (yAIRO). We believe it is important to enhance a constructive dialog between societies. The hope is to offer to young radiation oncologists a wealth of opportunities to refine their skills and gain access to the latest developments, according to a shared European vision.

## Introduction

The Young Italian Association of Radiotherapy and Clinical Oncology (yAIRO) represents the young radiotherapy community in Italy. It consists of Italian radiation oncologists and residents younger than 40 years. yAIRO’s primary aim is to develop and foster high-level educational, scientific and professional measures in the field of radiation oncology for young members. Considerable agreement has been reached in the yAIRO committee to support needs, priorities and expectations of residents and junior radiation oncologists at the early stages of their careers.

Among the others, the yAIRO steering committee has the responsibility to ensure that the scientific and educational proposals of the next generation of clinicians and scientists are carefully evaluated by the designated governance organs.

In line with the purpose of ESTRO Young, yAIRO provides a networking platform for young professionals; is responsible for the annual congress young session; collaborates with homologous national and international societies and plans educational activities and meetings.

With the intent to fully align yAIRO to the ESTRO 2030 vision [1], our ambition is to put the focus on the discipline and the profession in order to promote standards in radiation oncology for the large numbers of young members.

The start point is the ESTRO core set of the four “good” elements [2]: (i) good science, (ii) good education, (iii) good quality and (iv) good partnership. The main common denominator is to encourage links and fruitful connections between less experienced researchers and more experienced ones, to examine and discuss what are the potential roles and responsibilities of the radiation oncologists, from both clinical and academic backgrounds.

## Methods

The yAIRO committee includes six members: the coordinator, the past-coordinator and four councilors. The yAIRO committee has defined new inputs to refresh the content of yAIRO program with the aim to guarantee high-value radiation therapy landscape throughout the young Italian community. A first proposal was written after a literature revision until December 2020.

A virtual meeting was organized to discuss the initial document. At the end of the virtual meeting, feedback from all members was incorporated to create the final program.

The topics considered of highest importance were grouped in four focus groups, corresponding to the four “good” ESTRO elements [2]. Once yAIRO committee agreed with the final document, the resulting yAIRO statement was critically revised and approved by the Scientific Committee and the Board of the AIRO.

## yAIRO strategy

Although the number of new residents in radiation oncology is considerable every year in Italy (*n* = 134 in 2020) and young in-training professionals receive first year free AIRO membership in order to introduce them in our national society and scientific community.

Besides in-training members, also young board certified colleagues, are allowed to continue to take part to yAIRO activities, even if a relative low interest has been observed in the last years.

This issue has been specifically addressed by the yAIRO operational plan, with the aim to prioritize initiatives to increase young member’s participation in the society. There is a strong need to improve the communication channels between medical students/junior specialists and yAIRO.

This topic is particularly important to ensure a continuity in the on-going projects endorsed by yAIRO committee over the years and to disseminate the following initiatives as part of the future strategic development of the society.

### Good science

Scientific communication will be planned to offer an opportunity to refine skills and gain easy access to the latest developments in radiation therapy field.

The “yAIRO -day” is introduced: This will be a meeting specifically designed by young radiation oncologists for young radiation oncologists. It is a unique opportunity to share knowledge and experience in order to offer different perspectives to already existing tasks and to create a national network for knowledge sharing. It should be an opportunity to ask questions and enter a discussion on interdisciplinary and multidisciplinary approaches.

The “yAIRO -day” will be structured to guarantee plenty of time for discussion and to be highly interactive.

The core of this scientific day will comprise position papers from the annual AIRO meeting topics. Primary aim of this initiative is to address uncertainties and fears of young radiation oncologists, summarize the state of the art of the selected discussion topic and to look ahead to future directions for possible research and common development.

The “yAIRO -day” will also include a “medical student corner,” specifically organized to bring together the University community and radiation oncology world, to share information, discuss concerns and reflect on professional aims and perspectives.

### Good education

yAIRO recognizes the need and the complexity for an appropriate training in current radiation oncology. Set standards for education and clinical practice is paramount and requires complex coordination between different specialists. yAIRO recognizes the need to help to promote this process.

Information on core curricula will be collated for the specific needs of radiation oncology with the hope to develop a yAIRO Fellow program in the near future.

The start point is to create educational programs relevant to young members in their activities.

An important task of the yAIRO committee is to create a series of topic-oriented workshops.

These workshops will be part of a more articulated educational program and will cover topics of relevant scientific interest or educational need in clinical and research activities.

Topics are linked—but not limited—to: (i) how to structure and write a scientific paper for radiation oncology subspecialty, (ii) how to organize and fill in a clinical database, design a research project, apply for ethical approval and document results, (iii) assessment and management of work-related stress risk, (iv) share national and international experiences.

This format will give a valid opportunity to brainstorm freely and potentially build a network with in-training, young radiation oncologists and researchers throughout Italy.

In addition, each workshop’s group will be invited to submit a contribution that includes key points arising from the workshop, as well as current situation, perceived problems, research directions, next steps, potential recommendations. The format might be any combination of overview, editorials or systematic review.

The yAIRO online educational library will be further improved, expanded and promoted.

A dedicated yAIRO video program will be set up, aiming to provide educational online contents that show and discuss the availability and the cost-benefits of the radiotherapy technologies active across the different Italian departments, presenting the state of the art of our discipline in the country.

The hope is to open a new window to this direction with a useful and efficient hub of the students/young members’ needs and expectation across Italy, reducing technological distances and efficaciously transferring knowledge and facilitating career development.

Young members are invited to take an active part in the yAIRO initiatives: Their contribution can be addressed to the yAIRO Journal Club, that promotes the writing of reviews and comments on relevant, practice changing, scientific articles, or collaborating to the re-organization and update of the yAIRO website.

Furthermore, in order to improve the communication of news to the members, the participation of yAIRO on the main social media platforms is encouraged.

### Good quality

Advances in radiation oncology, both technological and clinical, have been extremely rapid in the last years and these changes have had a profound impact on the field. The development of scientific resources, including policy statements and documentation such as AIRO guidelines, core curricula, educational publications, conference proceedings and patients advocacy documents, requires a higher level of expertise. yAIRO will make every effort to reinforce analysis of scientific evidence and decision-making process in useful forms and actionable formats.

Clinical evidence is not static; therefore, an evolutionary process should be provided.

A documentation checklist, easily accessible on the website, should be useful to provide a quick-reference material with the latest news on consensus conferences, recommendations and key information. Strategic involvement of young members in specific committees, task groups and special meetings organized by the AIRO society will be promoted.

### Good partnership

At present, the concept of good partnership is strictly linked to multidisciplinary management. The increasingly complex multimodal treatments necessitate a close and a well-coordinated collaboration among specialists and to collaborate with other educational groups to increase multidisciplinary perspectives is therefore paramount. yAIRO is conscious of the importance of solid collaboration across disciplines and sectors. The purpose is to establish closer collaboration with other young national and international societies in the oncology field.

Steps will be made to promote this strategy.

yAIRO will facilitate its members to create collaborations, design clinical studies and support collaborative research in order to promote exchange of ideas, development of new inter- and intra-disciplinary networks and disseminate guidelines.

The magnitude of this task makes it unrealistic for yAIRO to tackle it alone and appropriate countermeasures to implement innovation, creativity, organization and open approach to data sharing should be taken at a national level to make it possible. An interdisciplinary vision, involving medical, statistical and social sciences, engineering and economics should be facilitated at best.

Finally, yAIRO could play a crucial role in bridging AIRO with the other medical societies to forge a strong partnership and improve the performance of the future AIRO members at national and international level.

## Conclusion

We believe that this document will assist all yAIRO members to collectively support the future priorities that will influence the direction of yAIRO’s activities at a national and international level in the forthcoming years.
